# A user-friendly software to easily count Anopheles egg batches

**DOI:** 10.1186/1756-3305-5-122

**Published:** 2012-06-19

**Authors:** Ali Mollahosseini, Marie Rossignol, Cédric Pennetier, Anna Cohuet, António dos Anjos, Fabrice Chandre, Hamid Reza Shahbazkia

**Affiliations:** 1 Departamento de Engenharia Eletrónica e Informática, Universidade do Algarve, 8008-139 Faro, Portugal; 2 Institut de Recherche pour le Développement, UMR MIVEGEC (UM1-UM2-CNRS 5290-IRD 224), 911 avenue Agropolis, B.P. 64501, 34394 Montpellier Cedex 5, France; 3 Institut de Recherche pour le Développement, UMR MIVEGEC (UM1-UM2-CNRS 5290-IRD 224), Contonou, Benin; 4 Centre de Recherche Entomologique de Cotonou, Ministry of Health, Cotonou, Benin; 5 Departamento de Ciências e Tecnologias, ISMAT - Instituto Superior Manuel Teixeira Gomes, 8500-508 Portimão, Portugal

## Abstract

**Background:**

Studies on malaria vector ecology and development/evaluation of vector control strategies often require measures of mosquito life history traits. Assessing the fecundity of malaria vectors can be carried out by counting eggs laid by Anopheles females. However, manually counting the eggs is time consuming, tedious, and error prone.

**Methods:**

In this paper we present a newly developed software for high precision automatic egg counting. The software written in the Java programming language proposes a user-friendly interface and a complete online manual. It allows the inspection of results by the operator and includes proper tools for manual corrections. The user can in fact correct any details on the acquired results by a mouse click. Time saving is significant and errors due to loss of concentration are avoided.

**Results:**

The software was tested over 16 randomly chosen images from 2 different experiments. The results show that the proposed automatic method produces results that are close to the ground truth.

**Conclusions:**

The proposed approaches demonstrated a very high level of robustness. The adoption of the proposed software package will save many hours of labor to the bench scientist. The software needs no particular configuration and is freely available for download on: http://w3.ualg.pt/∼hshah/eggcounter/.

## Background

Understanding the ecology and evolution of malaria vector species and populations is a key factor in controlling the disease they carry [1]. In several contexts, the estimation of the fitness of Anopheles mosquitoes is required. For instance, this may help to understand adaptation of different populations to given environmental conditions [2-4] or the opposite [5-7], interactions between the vectors and the parasites [8-12] or the vertebrate hosts [13]. More directly related to vector control, fitness measures can help deciphering the effect of insecticides [14], including new classes of insect growth regulators [15], effect of insecticide resistance [16,17], genetic manipulation of vector populations to make them resistant to parasites [18,19], or to impede their reproductive success. The fecundity is a key variable to assess the fitness of populations. In mosquitoes, it can be measured at several stages, but the number of eggs laid by females is often considered as a good estimate. Depending on the case study, the experiments may involve hundreds of females, possibly over many gonotrophic cycles and generations. As female Anopheles can lay over one hundred eggs every 2-3 days, one experiment only, often involves counting thousands of eggs [20,21]. Such a task is obviously tedious, time-consuming and prone to errors. These facts make the identification and counting of eggs a natural task for automation.

As software capable of correctly counting Anopheles eggs laid on filter paper was not available, we developed a fully automatic software together with its own user-friendly interface. Egg-Counter v1.0 has the capacity of counting individual eggs as well as eggs laid in piles. At first, it counts the eggs laid on the paper, excluding the debris. Then, the user is able to modify any details of the presented result by means of a provided user-friendly interface. The software was tested by comparing the automatic and manually corrected results with the number of manually counted eggs, and a precision of 98% was obtained. Egg-Counter is fully written in Java and, therefore, completely platform-independent; it can be downloaded, installed and used within a couple of minutes.

The rest of this paper is organized as follows: In Methods, the proposed method for processing the images is elaborated. A brief description of the software provided and its features are introduced in Software. Experimental results on 16 different images, taken in different experiments are presented in Results and Discussion.

## Methods

**Figure 1:**
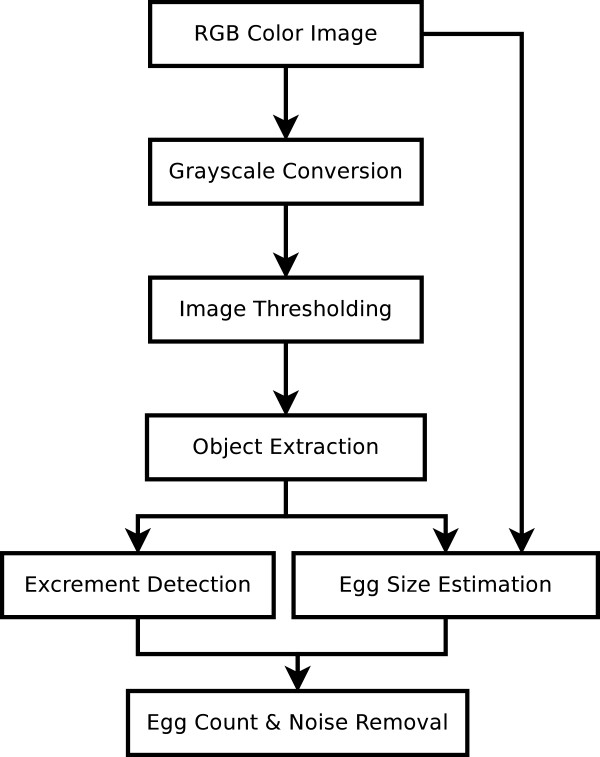
Proposed approach.

Figure 1 shows the diagram of the proposed algorithm for counting the eggs of an image. First the color image is converted to a grayscale by flattening the image. A binary map is extracted from the grayscale version of the image by means of image thresholding. Then, each connected component in the map is extracted as an object. To make the software as independent as possible from image resolutions and the camera settings, objects containing just one egg are detected and the size of one egg is estimated. Debris objects are detected and removed based on the original color image. Finally noise is removed and the number of eggs inside each of the remaining objects is calculated according to the estimated size of one egg.

### Grayscale Conversion

**Figure 2:**
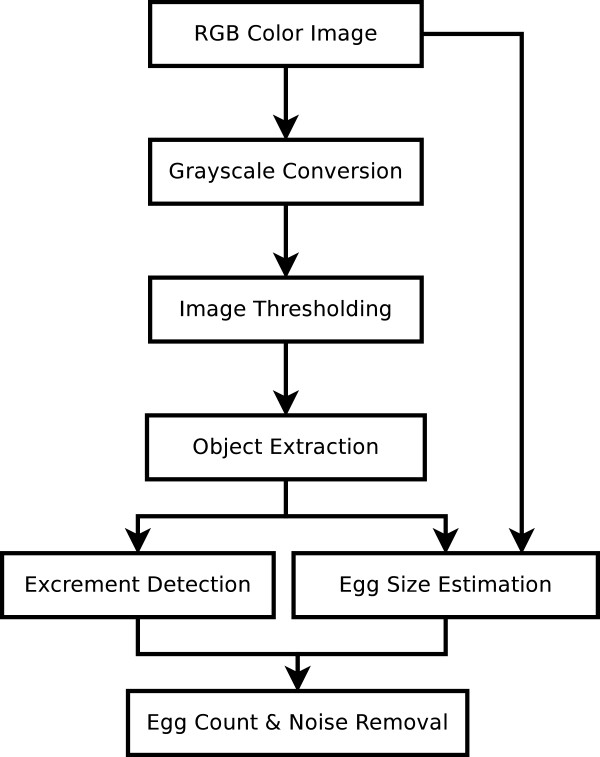
Sample image of the Anopheles eggs and resulting processed images.

Color images are converted to grayscale images. Luminance is typically computed as a weighed sum of the color components. The most often used weights were originally developed for encoding analog color television signals as:

(1) $$I_{i}=0.299\times R_{i}+0.587\times G_{i}+0.144\times B_{i},$$

where _*R* *i*_, _*G* *i*_ and _*B* *i*_ are the intensity levels of the red, green, and blue channels of the ^*i* *th*^pixel respectively. Figures 2(a) and 2(b) show a sample color image of the mosquitoes’ eggs and its resulting grayscale version.

### Image Thresholding

As shown in Figure 2(b), the eggs and debris are the dark objects on the white paper. Therefore, a proper binary map of the image can be used to distinguish them from the background. This binary map is defined as:

(2) $$B_{i}=\left\{\begin{array}{ll} 1, & if\;\;I_{i}\geq T_{\text{auto}}\\ 0, & \text{otherwise.} \end{array}\right)$$

Where _*B* *i*_ and _*I* *i*_ are the binary value and intensity of the ^*i* *th*^pixel respectively and _*T*auto_is the automatic threshold value based on an entropic thresholding method proposed in [22] as:

(3) $$\underset{T}{arg max}C_{B}(T)+C_{F}(T),$$

with

(4) $$C_{B}(T)=-\log\overset{T}{\underset{g=0}{\sum }}{\left(\frac{p(g)}{p(T)}\right)}^{2},$$

and

(5) $$C_{F}(T)=-\log\overset{T^{\prime \prime }}{\underset{g=T+1}{\sum }}{\left(\frac{p(g)}{1-p(T)}\right)}^{2}.$$

Where _*T*auto_ is the value of *T* that makes _*C* *B*_(*T*) + _*C* *F*_(*T*) maximum, *p*(*g*) is the probability of intensity level *g* and *p*(*T*) is the cumulative probability function. Unlike the original method [22], here $T^{\prime \prime }$ controls the maximum value of threshold in order to remove high exposure in the images caused by flash where empirically $T^{\prime \prime }\in [160,180]$. Figure 2(c) shows the resulting binary map of the sample image. As it is shown eggs and debris are properly extracted, after the automatic threshold is found.

### Object Extraction

In this step eggs and debris are detected by extracting eight-connected neighborhood components. Putting it simply, connected component labeling is the process of assigning labels to the foreground elements in such a way that adjacent foreground elements are assigned to the same label [23]. Here, adjacent means 8-adjacent pixels neighborhood.

### Egg Size Estimation

Usually, a single mosquito egg has an elongated ellipse shape, while a pile of eggs looks more circular (see Figure 2(b)). Therefore, the eccentricity of the object is used to distinguish single eggs from the piles of eggs. Eccentricity measures how much an object deviates from being circular. It is the ratio of the distance between the foci and its major axis length [24], defined as:

(6) $$e=\sqrt{1-\frac{\lambda_{1}}{\lambda_{2}}},$$

(7) $$\lambda_{i}=\frac{{\mu^{\prime \prime }}_{20}+{\mu^{\prime \prime }}_{02}}{2}\pm \frac{\sqrt{4{\mu^{\prime \prime }}_{11}+{\left({\mu^{\prime \prime }}_{20}-{\mu^{\prime \prime }}_{02}\right)}^{2}}}{2}.$$

Where ${\mu^{\prime \prime }}_{\mathrm{mn}}$ is the central moment of order (*mn*), defined as:

(8) $$\mu_{\mathrm{mn}}^{\prime \prime }=\underset{\left(u,v\right)\in R}{\sum }{\left(u-\bar{x}\right)}^{p}{\left(v-\bar{x}\right)}^{q},$$

where $\left(\bar{x},\bar{y}\right)$ is the centroid of the object and $\left(u,v\right)\in R$ represents whole set of pixel coordinates inside the object. The value of the eccentricity falls in the interval [0,1]. A value of one indicates a perfect circle and zero indicates a line segment. In this step, the objects with high eccentricity, _*e*1_<*e*<_*e*2_, are selected as eggs. Values of _*e*1__*e*2_can be set from 0.95 up to 0.99. Experimental results show that more than 80% of the components containing one egg are in this range. Finally, the mode of the size of the candidate objects is chosen as the estimated size for one egg.

### Debris Detection

Debris are black regions extracted on the binary map (see Figure 2(b) and 2(c)) that could be assessed as eggs on the binary map. Nevertheless, a yellow brownish area often presents around the regions of debris (see Figure 2(a)) that can be used to detect debris. For extracting the brownish area near the debris, a chroma value for each pixel is defined as:

(9) $$C_{i}=\max(R_{i},G_{i},B_{i})-\min(R_{i},G_{i},B_{i}).$$

In this step, the objects that have at least *X* pixels with *C*>_*C* *E*_ in their radii *r* are considered as debris. Parameter _*C* *E*_is 0.15 by default, but the application allows the user to tune it, resulting in a change of the detection sensitivity. Because eggs have a dark color and the paper is white, the rate of true negatives (considering non-debris areas as debris) is negligible and it only happens for the eggs that are inside the yellow brownish area.

### Egg Count and Noise Removal

The number of the eggs *N* inside the object *i*, is estimated as:

(10) $$N(i)=\left\{\begin{array}{ll} ∥\frac{A(i)}{S}∥, & A(i)\leq 2\times S\\ ∥\frac{A(i)\times C}{S}∥, & \text{otherwise,} \end{array}\right)$$

where *A*(*i*) is the area of the object, *S* is the estimated size of one egg, ∥.∥ is the round function and *C* is the accumulative ratio which should be less than one. The accumulative ratio is necessary due to the fact that regions in the intersection of the eggs inside a pile are shadowed by the eggs and are usually considered as a connected object in the binary map. Therefore, multiplying by this ratio allows better estimation of the number of eggs.

It should be mentioned that thresholding may produce noise, especially in the grid lines of the paper. There are many methods for noise reduction such as low-pass filters and other smoothing operators. However, these methods highly depend on the size of the filter. Here components smaller than half of the estimated size of one egg, are considered as noise in the Equation (10), and are removed.

## Software

**Figure 3:**
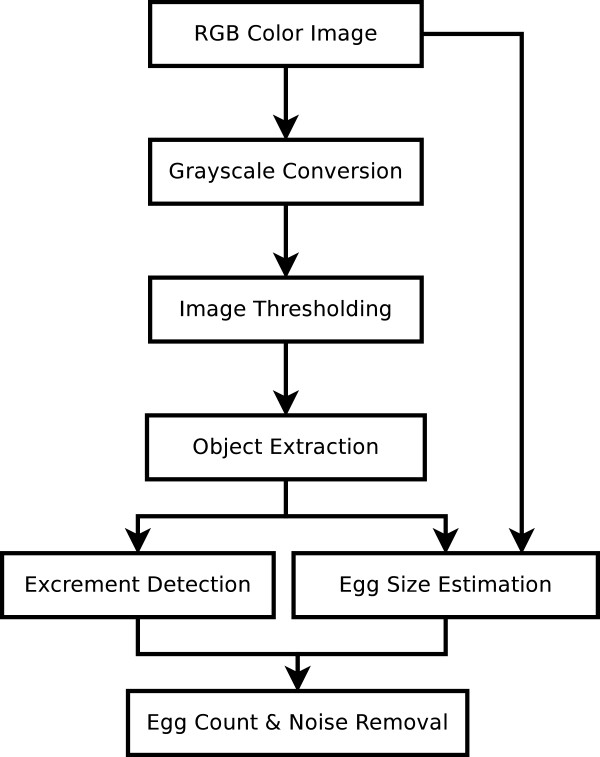
**Basic graphical user interface.** Zoom utility signaled by ‘a’, and egg count by ‘b’.

The algorithm explained above, has been implemented in Java as open source software. After the user opens an image, the software automatically computes the threshold value, detects and removes areas of debris, estimates the number of eggs inside each pile of the eggs and removes the noise. After that, the user is able to remove, add, and edit the automatically detected features by means of a user-friendly graphical interface. Figure 3 shows the basic graphical user interface of the software. The user can zoom, select single and/or multiple objects, modify their properties etc. and after each modification the total number of eggs is recalculated.

**Figure 4:**
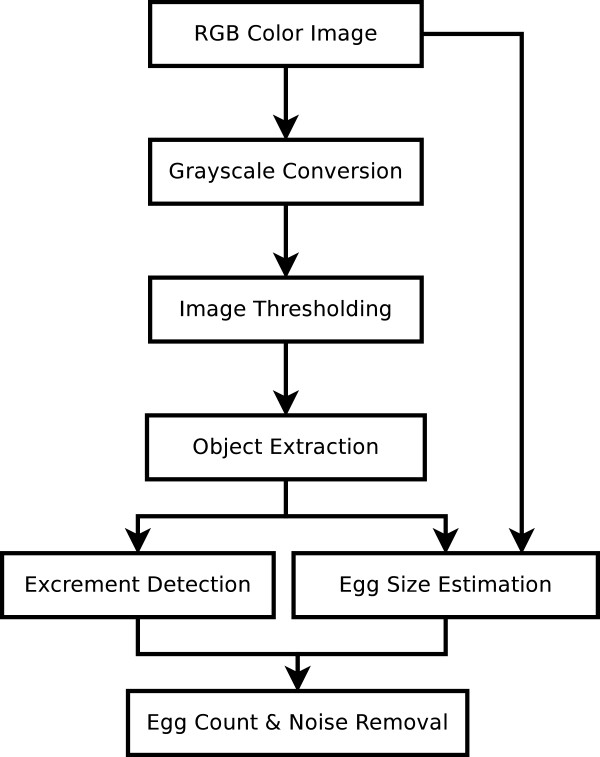
**Advanced graphical user interface.** ‘c’ signals the advanced features for changing the automatic threshold and chroma value. ‘b’ signals a manually corrected egg.

Figure 4 shows advanced options of the software, which allows the user to change the sensitivity of the thresholding as well as the sensitivity of the debris detection process. Finally, the user can save the results as an image indicating the number of eggs beside each object as well as the total number of eggs. Figure 2(d) shows a sample output of the software, and Figure 2(e) shows the result after a small manually modification. The application estimated 103 eggs and, after manual modification, it showed 92 eggs. Corrections are mainly required on the debris areas without enough colorful pixels beside them, and eggs that fall in the neighborhood of debris areas.

## Results and Discussion

The software was tested over 16 randomly chosen images from 2 different experiments. Table 1 shows the number of the eggs estimated by the software; the result after manually correction; the ground truth; the time that the application took in seconds; and the average time for manually counting in minutes.

**Table 1:** Results of 16 different images

					
Image	Ground Truth/	Auto./	Mouse	Output/	Missed
Ref.	MINUTES	Sens.	Clicks	SECONDS	Eggs
01	702 / 29	707	4	711 / 9	1%
02	342 / 20	320	8	366 / 18	7%
03	608 / 40^#^	399 / 620	1^1*^	620 / 35	2%
04	391 / 21	277 / 400	1^2*^	400 / 35	2%
05	372 / 21	314 / 363	0^3*^	363 / 25	2%
06	219 / 20	212	0	219 / 18	0%
07	460 / 27	478	3	455 / 20	1%
08	086 / 15	102	2	89 / 18	3%
09	114 / 20	118	1	119 / 18	4%
10	280 / 21	300	5	272 / 20	2%
11	352 / 25	342	5	360 / 20	2%
12	480 / 25	701	2	489 / 20	2%
13	022 / 06	23	1	22 / 15	0%
14	297 / 20	267	19	297 / 115	0%
15	331 / 21	358	9	331 / 110	0%
16	311 / 20	302	9	311 / 110	0%

^#^Automatic sensitivity failed and the software needed to be restarted.

^∗^Results after correction of sensitivity.

Column “Auto. / Sens.” presents both the automatically detected eggs using the default parameters and after adjusting the sensitivity parameter.

As it is shown in the error rate column, the precision of the software after a first correction is on average more than 98%, whereas the time saved is enormous. For image number 3, the automatic processing was very poor and the user had to manually set the sensitivity parameters. It should be mentioned that a proper sensitivity can be easily found by just adjusting a sliding bar. Manually counting all eggs of an image containing more than 500 eggs can take up to 30 minutes but, the same image, can be processed by Egg-Counter v1.0 in only a few seconds.

The results show that the proposed automatic method produces results that are close to the ground truth, and by making a few adjustments, mainly in the regions of debris and the paper’s grid lines, these results get even closer to the ground truth. Multiplying the time gained by the number of images and experiments, results in economizing an extremely large amount of time. The sensitivity value of the test results for assessing the eggs is around 0.945 in the fully automatic use of the software and is higher after a few mouse clicks. The specificity value, in this case, makes no sense since the true negatives are not actually known. Elimination of thousands of noise pixels during different stages of the analysis does not allow a reliable calculation of the specificity of the software. Positive predictive value of the test is set around 0.921 for the full automatic version.

The proposed algorithm works well, if eggs are clearly darker than the background paper, even if they are brown or another dark color. To introduce better and higher contrast and a wider color difference between the paper and laid eggs, we suggest using white or semi-white paper. In addition, it is highly recommended to take images with a camera of at least 5M pixels, using the lowest ISO and maximum shutter speed and avoiding re-sizing the images afterwards. Depending on your Laboratory lightning conditions, you might need to use a flash.

## Conclusion

In this article, a promising new software package for automatically counting Anopheles eggs was presented. A very intuitive graphical user interface is available, that allows correction of the detection results, if needed. Additionally, in the parameters section of the interface it is possible to control the sensitivity to egg detection. Nevertheless, the proposed approaches involved in the processing of such images demonstrated a very high level of robustness, as it was shown by the results. The adoption of the proposed software package will save many hours of labor for the bench scientist.

## Competing interests

The authors declare that they have no competing interests.

## Author’s contributions

Ali Mollahosseini, António dos Anjos and Hamid Reza Shahbazkia contributed with the proposed methods and software and Marie Rossignol, Cédric Pennetier, Anna Cohuet and Fabrice Chandre for testing and providing the necessary material. All authors read and approved the final version of the manuscript.

## References

[B1] Ferguson HM Dornhaus A Beeche A Borgemeister C Gottlieb M Mulla MS Gimnig JE Fish D Killeen GF Ecology: A prerequisite for malaria elimination and eradication PLoS Med 2010 7 8 e1000303 20689800 10.1371/journal.pmed.1000303 PMC2914634

[B2] Yaro AS Dao A Adamou A Crawford JE Traoré SF Touré AM Gwadz R Lehmann T Reproductive output of female Anopheles gambiae (Diptera: Culicidae): comparison of molecular forms J Med Entomol 2006 43 5 833 839 17017216 10.1603/0022-2585(2006)43[833:roofag]2.0.co;2

[B3] Sharma A Parasher H Singh OP Adak T Species B of Anopheles culicifacies (Diptera: Culicidae) is reproductively less fit than species A and C of the complex Acta Tropica 2009 112 3 316 319 19679093 10.1016/j.actatropica.2009.08.006

[B4] Hogg JC Thomson MC Hurd H Comparative fecundity and associated factors for two sibling species of the Anopheles gambiae complex occurring sympatrically in The Gambia Med Vet Entomol 1996 10 4 385 391 8994142 10.1111/j.1365-2915.1996.tb00761.x

[B5] Manda H Gouagna LC Foster WA Jackson RR Beier JC Githure JI Hassanali A Effect of discriminative plant-sugar feeding on the survival and fecundity of Anopheles gambiae Malaria Journal 2007 6 113 124 17711580 10.1186/1475-2875-6-113 PMC2034389

[B6] Gary RE Foster WA Effects of available sugar on the reproductive fitness and vectorial capacity of the malaria vector Anopheles gambiae (Diptera: Culicidae) J Med Entomol 2001 38 22 28 11268686 10.1603/0022-2585-38.1.22

[B7] Pfaehler O Oulo DO Gouagna LC Githure J Guerin PM Influence of soil quality in the larval habitat on development of Anopheles gambiae Giles J Vector Ecol 2006 31 2 400 405 17249359 10.3376/1081-1710(2006)31[400:iosqit]2.0.co;2

[B8] Yaro AS Touré AM Guindo A Coulibaly MB Dao A Diallo M Traoré SF Reproductive success in Anopheles arabiensis and the M and S molecular forms of Anopheles gambiae: Do natural sporozoite infection and body size matter? Acta Tropica 2012 122 87 93 22198241 10.1016/j.actatropica.2011.12.005

[B9] Hogg JC Hurd H The effects of natural Plasmodium falciparum infection on the fecundity and mortality of Anopheles gambiae s. l. in north east Tanzania Parasitology 1997 114 Pt4 325 331 9107019 10.1017/s0031182096008542

[B10] Hogg JC Hurd H Malaria-induced reduction of fecundity during the first gonotrophic cycle of Anopheles stephensi mosquitoes Med Vet Entomol 1995 9 2 176 180 7787226 10.1111/j.1365-2915.1995.tb00175.x

[B11] Ferguson HM Read AF Why is the effect of malaria parasites on mosquito survival still unresolved? Trends in Parasitology 2002 18 6 256 261 12036738 10.1016/s1471-4922(02)02281-x

[B12] Ahmed AM Hurd H Immune stimulation and malaria infection impose reproductive costs in Anopheles gambiae via follicular apoptosis Microbes and Infection 2006 8 2 308 315 16213176 10.1016/j.micinf.2005.06.026

[B13] Lyimo IN Keegan SP Ranford-Cartwright LC Ferguson HM The impact of uniform and mixed species blood meals on the fitness of the mosquito vector Anopheles gambiae s.s: does a specialist pay for diversifying its host species diet? Journal of Evolutionary Biology 2012 25 3 452 460 22221693 10.1111/j.1420-9101.2011.02442.x

[B14] Mosqueira B Duchon S Chandre F Hougard JM Carnevale P Mas-Coma S Efficacy of an insecticide paint against insecticide-susceptible and resistant mosquitoes - part 1: laboratory evaluation Malaria Journal 2010 9 340 346 21108819 10.1186/1475-2875-9-340 PMC3001744

[B15] Dhadialla TS Carlson GR Le DP New insecticides with ecdysteroidal and juvenile hormone activity Annu Rev Entomol 1998 43 545 569 9444757 10.1146/annurev.ento.43.1.545

[B16] Okoye PN Brooke BD Hunt RH Coetzee M Relative developmental and reproductive fitness associated with pyrethroid resistance in the major southern African malaria vector, Anopheles funestus Bulletin of Entomological Research 2007 97 6 599 605 17997873 10.1017/S0007485307005317

[B17] Djogbénou L Labbé P Chandre F Pasteur N Weill M Ace-1 duplication in Anopheles gambiae: a challenge for malaria control Malaria Journal 2009 8 70 76 19374767 10.1186/1475-2875-8-70 PMC2679766

[B18] Li C Marrelli MT Yan G Jacobs-Lorena M Fitness of transgenic Anopheles stephensi mosquitoes expressing the SM1 peptide under the control of a vitellogenin promoter J Hered 2008 99 3 275 282 18334506 10.1093/jhered/esn004 PMC4154370

[B19] Lambrechts L Koella JC Boëte C Can transgenic mosquitoes afford the fitness cost? Trends in Parasitology 2008 24 4 7 18164248 10.1016/j.pt.2007.09.009

[B20] Munhenga G Brooke BD Chirwa TF Hunt RH Coetzee M Govender D Koekemoer LL Evaluating the potential of the sterile insect technique for malaria control: relative fitness and mating compatibility between laboratory colonized and a wild population of Anopheles arabiensis from the Kruger National Park, South Africa Parasites & Vectors 2011 4 208 11 22041133 10.1186/1756-3305-4-208 PMC3216276

[B21] Kweka EJ Owino EA Mwang’onde BJ Mahande AM Nyindo M Mosha F The role of cow urine in the oviposition site preference of culicine and Anopheles mosquitoes Parasites & Vectors 2011 4 184 190 21943071 10.1186/1756-3305-4-184 PMC3193820

[B22] Yen JC Chang FJ Chang S A new criterion for automatic multilevel thresholding Image Processing, IEEE Transactions on 1995 4 3 370 378 10.1109/83.366472 18289986

[B23] González R Woods R 2001 Addison-Wesley Longman Publishing Co., Inc., Boston, MA, USA

[B24] Burger W Burge MJ Digital Image Processing: an algorithmic introduction using Java 2008 Springer, New York

